# Biological Activities of Selected Plants and Detection of Bioactive Compounds from *Ardisia elliptica* Using UHPLC-Q-Exactive Orbitrap Mass Spectrometry

**DOI:** 10.3390/molecules25133067

**Published:** 2020-07-06

**Authors:** Pei Lou Wong, Nurul Azila Fauzi, Siti Norhamimah Mohamed Yunus, Nur Ashikin Abdul Hamid, Siti Zulaikha Abd Ghafar, Awanis Azizan, Nur Khaleeda Zulaikha Zolkeflee, Faridah Abas

**Affiliations:** 1 Department of Food Science, Faculty of Food Science and Technology, Universiti Putra Malaysia, Serdang 43400, Selangor, Malaysia; plou_wong@hotmail.com (P.L.W.); azilafauzi9520@gmail.com (N.A.F.); cikmiemayunus@gmail.com (S.N.M.Y.); ctzue.agb@gmail.com (S.Z.A.G.); 2 Laboratory of Natural Products, Institute of Bioscience, Universiti Putra Malaysia, Serdang 43400, Selangor, Malaysia; shikinhamid89@yahoo.com (N.A.A.H.); awanis_azizan@yahoo.com (A.A.); khaleeda_zulaikha@yahoo.com (N.K.Z.Z.)

**Keywords:** underutilized plants, antioxidant, free radical scavenging, anti-α-glucosidase, phytochemical characterization

## Abstract

Plants and plant-based products have been used for a long time for medicinal purposes. This study aimed to determine the antioxidant and anti-α-glucosidase activities of eight selected underutilized plants in Malaysia: *Leucaena leucocephala, Muntingia calabura, Spondias dulcis, Annona squamosa, Ardisia elliptica, Cynometra cauliflora, Ficus auriculata*, and *Averrhoa bilimbi.* This study showed that the 70% ethanolic extract of all plants exhibited total phenolic content (TPC) ranging from 51 to 344 mg gallic acid equivalent (GAE)/g dry weight. *A. elliptica* showed strong 2,2-diphenyl-1-picrylhydrazyl (DPPH) and nitric oxide (NO) scavenging activities, with half maximal inhibitory concentration (IC_50_) values of 2.17 and 49.43 μg/mL, respectively. Most of the tested plant extracts showed higher inhibition of α-glucosidase enzyme activity than the standard, quercetin, particularly *A. elliptica, F. auriculata*, and *M. calabura* extracts with IC_50_ values of 0.29, 0.36, and 0.51 μg/mL, respectively. A total of 62 metabolites including flavonoids, triterpenoids, benzoquinones, and fatty acids were tentatively identified in the most active plant, i.e., *A. elliptica* leaf extract, by using ultra-high-performance liquid chromatography (UHPLC)–electrospray ionization (ESI) Orbitrap MS. This study suggests a potential natural source of antioxidant and α-glucosidase inhibitors from *A. elliptica*.

## 1. Introduction

Malaysia is a country that is recognized for its diverse flora and fauna. Various species of plants, animals, and microorganisms offer Malaysians an extensive source of nutritious food and medicines. Furthermore, the antioxidant activities of different parts of plants, including roots, leaves, stalks, flowers, fruits, and seeds were studied. Acknowledgement of the potential medicinal benefits of local plants along with the development of modern technology motivated researchers, pharmacists, and physicians to explore Malaysian biodiversity. In addition to commonly consumed local herbs and fruits, some underutilized local species have the potential to act as alternative sources of micronutrients, vitamins, and health-promoting secondary plant metabolites [1]. These species include “petai belalang” (*Leucaena leucocephala* (Lam.) de Wit (Fabaceae))*,* “ceri hutan” (*Muntingia calabura* L. (Muntingiaceae)), “kedondong” (*Spondias dulcis* Parkinson (Anacardiaceae)), “nona” (*Annona squamosal* L. (Annonaceae)), “mata ayam” (*Ardisia elliptica* Thunb. (Primulaceae)), “katak puru” (*Cynometra cauliflora* L. (Fabaceae)), “ara” (*Ficus auriculata* Lour. (Moraceae)), “belimbing buluh” (*Averrhoa bilimbi* L. (Oxalidaceae)), and others. However, the biological activity and the chemical profile of these underutilized plants remain unknown. Therefore, this study was conducted to fill in the current research gap existing for these plants.

The prevalence of Malaysian adults suffering from diabetes mellitus increased from 11.6% in 2006 to 15.2% in 2011; the rate is projected to reach 21.6% by 2020 [2]. Previous studies showed correlations between oxidative stress and diabetes [3]. Human bodies rely on endogenous and exogenous antioxidants to minimize the cellular damage and stress caused by free radicals by maintaining redox balance. Bouayed and Bohn [4] stated that antioxidants from fruits, vegetables, and other sources play a significant role in assisting the endogenous antioxidant defense system, which includes superoxide dismutase, catalase, and glutathione peroxidase, in preventing oxidative stress.

Diabetic patients suffer from an abnormal increase of blood glucose level after meal ingestion, a condition commonly known as postprandial hyperglycemia. α-Glucosidase, which is located in the epithelium of the small intestine, is one of the enzymes responsible for carbohydrate digestion. Postprandial hyperglycemia can be reduced through several means such as by suppressing α-glucosidase activity, thereby delaying the carbohydrate hydrolysis and glucose absorption by the cells [5]. Triggle and Ding [6] reported that synthetic drugs, such as metformin, sulfonylureas, thiazolidinediones, and other α-glucosidase inhibitors (including acarbose and miglitol, which were introduced as treatment for diabetes and are also known for their undesirable side effects) increased cardiovascular risk and induction of hepatotoxicity. Since modern medical treatments encourage the use of plant-based functional foods and drugs, particularly in diabetes treatment, numerous studies were conducted in the quest for effective hypoglycemic agents. Kumar et al. [7] suggested that natural α-glucosidase inhibitors from plant sources, including flavonoids, alkaloids, terpenoids, anthocyanins, glycosides, and phenolic compounds, are effective in inhibiting the activity of α-glucosidase. Therefore, this study aimed to determine the total phenolic content (TPC), as well as antioxidant (2,2-diphenyl-1-picrylhydrazyl (DPPH) and nitric oxide (NO) free radical scavenging) and anti-α-glucosidase activities, of the leaves of selected underutilized Malaysian plants. This study provides the first detailed metabolite profile of the most active extract, i.e., *Ardisia elliptica*, by using ultra-high-performance liquid chromatography (UHPLC)-electrospray ionization (ESI) Orbitrap MS.

## 2. Results and Discussion

### 2.1. Total Phenolic Content (TPC) of the Selected Plant Extracts

The current study showed that all leaf extracts had high TPC concentrations ranging from 50.90 ± 0.69 to 344.17 ± 10.80 mg gallic acid equivalent (GAE)/g crude extract (Table 1). The leaf extract of *C. cauliflora* had the highest phenolic content, followed by that of *A. elliptica* and *A. squamosa* (253.10 ± 1.19 and 199.62 ± 7.40 mg GAE/g crude extract, respectively), while the leaf extract of *S. dulcis* had the lowest phenolic content. A lower TPC value for *S. dulcis* was also reported by Rahman et al. [8]. Unlike other species from the *Spondias* family, this particular species was not thoroughly studied, probably due to its low phenolic content. The TPCs of *L. leucocephala, M. calabura*, and *F. auriculata* were not significantly different (*p* > 0.05), with values of 175.75 ± 3.48, 172.32 ± 3.39, and 167.15 ± 2.04 mg GAE/g crude extract, respectively, followed by the leaf extract of *A. bilimbi* at 97.50 ± 3.46 mg GAE/g crude extract. Variations in the applied extraction system might influence the phenolic content evaluated in plant extracts. Ethanol was believed to be able to extract more phenolic compounds compared to acetone, water, and methanol [9]. Methanolic *A. squamosa* and *C. cauliflora* leaf extracts were reported to have lower TPC compared to current study [10,11], while the 50% ethanolic *M. calabura* extract was found to retain higher TPC compared to absolute ethanol and water extracts [12]. Meanwhile, soaking of *A. elliptica* leaves in 95% methanol yielded a lower TPC compared to the present study which employed sonication-assisted extraction [13]. Furthermore, soaking of *A. bilimbi* leaves in 70% ethanol was found to result in higher TPC compared to the current extract [14]. Despite the choice of organic solvents used and the water content present in the extraction, the level of phenolic compounds produced in plant tissue might be affected by environmental factors, climatic factors, and soil nutrients [15].

### 2.2. DPPH and NO Free Radical Scavenging Activity of the Selected Plant Extracts

Two in vitro antioxidant assays were used to investigate the antioxidant potential of selected leaf extracts, which using DPPH and NO free radical scavenging assays. *A. elliptica* and *C. cauliflora* leaf extracts showed stronger antioxidant capability than the standard quercetin in the DPPH assay, whereas none of the leaf extracts showed higher activity than the standards quercetin and gallic acid in the NO assay. In addition, no similar trend was observed in free radical scavenging activities when the DPPH and NO assays were compared (Table 1). *A. elliptica*, *C. cauliflora*, and *M. calabura* extracts showed high activity in DPPH assay with half maximal inhibitory concentration (IC_50_) values of 2.17 ± 0.08, 2.88 ± 0.05, and 4.67 ± 0.21 µg/mL, respectively. On the other hand, the plant extracts that showed strong capability in scavenging NO radicals were *A. elliptica* and *M. calabura* extracts, with IC_50_ values of 49.43 ± 0.18 and 59.40 ± 3.39 µg/mL, respectively. However, *C. cauliflora* showed weak inhibition in the NO assay, with an IC_50_ value of 118.62 ± 3.44 µg/mL. This might be due to the slight difference in mechanism between both assays. DPPH radicals were scavenged by antioxidants that act as a hydrogen donor [16]. On the other hand, two possible pathways are available to scavenge NO radicals: one is the removal of hydrogen from antioxidants, and the other is by receiving a single-electron transfer from the NO radical to form an antioxidant cation, followed by an oxidation process [17].

Despite the divergence of mechanism of both antioxidant assays, the current study showed that *A. elliptica* leaf extract had the greatest antioxidant potential among all tested plants in the DPPH and NO scavenging assays, with IC_50_ values of 2.17 ± 0.08 and 49.43 ± 0.18 µg/mL, respectively (Table 1). Pearson correlation was employed to evaluate the association between TPC and antioxidant activities of the active extracts. *A. elliptica* and *C. cauliflora* demonstrated week and moderate positive correlation between TPC and the DPPH assay with *R* values of 0.27 and 0.46, respectively. *M. calabura* showed strong positive correlation (*R* value = 0.87), which suggests that phenolic compounds in *M. calabura* contribute to the DPPH assay as reported by previous study [12]. Furthermore, it is worth to note that *C. cauliflora* demonstrated a strong negative correlation with *R* value = −0.99 between TPC and the NO assay. Other types of metabolites such as tannin, terpenoids, and saponins found in *C. cauliflora* leaves might provide a positive effect on the bioactivities [18]. In addition, *A. elliptica* showed a strong positive correlation with *R* value = 0.87 between TPC and the NO assay, implying that secondary metabolites as phenolic compounds may be responsible for the plants’ antioxidant capability, which supported the corresponding high TPC values in *A. elliptica* leaf extract [4,19].

### 2.3. Anti-α-Glucosidase Activity of the Selected Plant Extracts

Among the tested plant samples, *S. dulcis* showed the weakest anti-α-glucosidase activity followed by *A. bilimbi*, with IC_50_ values of 45.52 ± 2.18 and 26.91 ± 0.58 µg/mL, respectively (Table 1). The remaining leaf extracts had potent enzyme inhibition activity with IC_50_ values between 0.29 ± 0.01 and 6.62 ± 0.19 µg/mL. The IC_50_ values shown by most of the leaf extracts were equal to or lower than that of the positive control, quercetin, suggesting the potential for the use of plant-based material as an anti-diabetic agent. *A. elliptica*, *F. auriculata*, *M. calabura*, and *C. cauliflora* leaf extracts showed high activity for the anti-α-glucosidase enzymatic reaction without a statistical difference (*p* > 0.05), with IC_50_ values ranging from 0.29 ± 0.01 to 0.90 ± 0.02 µg/mL. Pearson correlation analysis showed that the *C. cauliflora* extract demonstrated strong positive correlation between TPC and anti-α-glucosidase activity (*R* = 0.84), while *A. elliptica* and *M. calabura* extracts showed moderate positive correlation with *R* values of 0.71 and 0.59, respectively. The high content of phenolic compounds could contribute to the activity of these plants owing to the notable high TPC observed in the current study [20]. Apart from phenolic compounds, the presence of plant steroids, triterpenoids, and other nitrogenous compounds was also responsible for anti-diabetic effects [7]. The complexity of plant metabolites anticipated the simultaneous interactions of various phytoconstituents either synergistically or antagonistically on the bioactivities [21]. Therefore, the findings of this study further strengthen previous claims regarding the anti-diabetic potential of these plants.

### 2.4. UHPLC–MS/MS Analysis of Ardisia Elliptica

**Figure 1:**
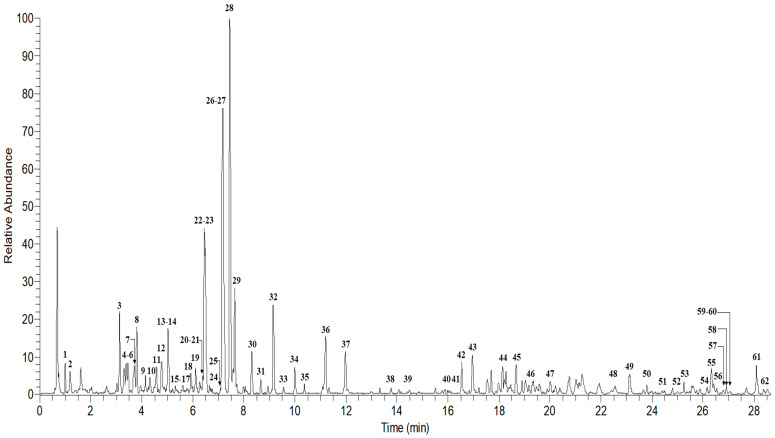
Total ion chromatogram of 70% ethanolic leaves extract of *A. elliptica.*

Based on biological activity results, *A. elliptica* was identified as the most potent extract that showed antioxidant and anti-α-glucosidase activities. In view of the limited published information regarding the metabolite profile, further phytochemical characterization of the active extract was performed by using UPLC-ESI-Orbitrap MS/MS. A total of 62 metabolites were tentatively characterized in *A. elliptica* leaf extract (Table 2). The identification of the metabolites was based on the comparison with the literature and several online mass databases (Knapsack, Metabolomics Workbench, Human Metabolome Database, PubChem, and MassBank). The total ion chromatogram showed the major components observed between 0 and 30 min (Figure 1). Flavonoids accounted for 63% of the 62 tentatively identified metabolites, which may contribute to the high TPC value of *A. elliptica* leaf extract. Flavonoids such as quercetin, kaempferol, myricetin, and catechin derivatives, which are known to be abundant in plant matrices, could be identified in *A. elliptica* leaf extract. Other types of metabolites identified include fatty acid derivatives, benzoquinones, triterpenoids, phenolic lipids, and phenol ester.

A total of 10 metabolites were tentatively identified as quercetin derivatives in *A. elliptica* leaf extracts, with the signature aglycone fragment ion at *m/z* 301 (peak **33**) and the characteristic fragment ions at *m/z* 271 and 151 in their MS/MS spectra [20]. Peak **7** was tentatively identified as quercetin 3-*O*-(2″-*O*-galloyl)-rutinoside with a deprotonated molecule at *m/z* 761.1343 [M − H]^−^. Further fragmentation analysis of the compound showed fragmentation ions at *m/z* 610, 301, and 169, indicating the presence of rutin, quercetin, and gallic acid units. Peak **15** showed a deprotonated molecule at *m/z* 755.2025 and a fragment ion at 301, showing the loss of two rhamnosyl and one glucosyl moieties. Peak **15** was provisionally identified as quercetin 3-*O*-(2,6-di-*O*-rhamnosylglucoside) according to the comparison of previous MS/MS data [19]. Data presented in Table 2 indicated that peak **18** with *m/z* 595.1293 yielded fragment ion at *m/z* 301 attributed to the quercetin aglycone formed by the loss of a lathyrosyl residue (294 u). Meanwhile, peak **21** was tentatively identified as rutin with a deprotonated molecule at *m/z* 609.1450 and gave quercetin aglycone at *m/z* 301 due to the loss of a rutinoside residue (308 u). Similar to peak **23** at *m/z* 380.9911, loss of a sulfate residue (80 u) yielded a fragment ion at *m/z* 301. Peaks **24**, **25**, and **28** were identified as quercetin-3-*O*-glucoside, quercetin-3-*O*-arabinoside, and quercetin-3-*O*-rhamnoside, based on the deprotonated molecules at *m/z* 463.0875, 433.0768, and 447.0917, respectively. Based on the mass spectrum, the radical aglycone anion (*m/z* 300) also could be observed from the regular homolytic cleavage of glycosidic bonds in the deprotonated quercetin ion (*m/z* 301) by negative ion mode. The relative abundance of radical anions is affected by the collision energy with a relative increase in collision energy leading to a relative increase of radical anion [22]. Peak **20** was conditionally identified as quercetin 3-*O*-α-l-rhamnoside-7-*O*-β-d-glucoside with a deprotonated molecule at *m/z* 609.1451 with fragmentation ions at *m/z* 463, 447, and 301, which resulted from the removal of rhamnosyl residue (146 u) and glucose (162 u) moieties [19].

Peaks **16**, **17**, **19**, and **22** were identified as myricetin derivatives based on the presence of fragmentation ion at *m/z* 316, which corresponds to the myricetin aglycone fragment in the MS/MS spectra [19]. Peaks **16**, **17**, **19**, and **22** were tentatively assigned as myricetin-3-*O*-glucoside, myricetin-3-*O*-rutinoside, myricetin-3-*O*-arabinoside, and myricetin-3-*O*-rhamnoside, based on the deprotonated molecules at *m/z* 479.0816, 625.1396, 449.0713, and 463.0869 corresponding to the loss of sugar moieties, respectively. The radical myricetin anion (*m/z* 316) observed instead of *m/z* 317 might be due to the scission of glycosidic bonds as a result of high collision energy in the system [22].

Peak **13** showed a deprotonated molecule at *m/z* 289.1802 with fragment ions at *m/z* 245, 179, and 137, suggesting that the presence of catechin corresponded to the reported data [19]. Peak **3** was tentatively identified as catechin 6-*C*-glucoside based on deprotonated molecule at *m/z* 451.3394 and gave fragment ion at *m/z* 289 attributed to catechin aglycone formed by the loss of a glucosyl moiety (162 u). Epigallocatechin-3-gallate and its isomer were assigned as peaks **14** and **31** at *m/z* 457.0766 based on fragment ions observed at *m/z* 305 and *m/z* 169, which correspond to epigallocatechin and gallic acid moieties, respectively.

Peaks **26, 30**, and **34** were identified as kaempferol derivatives. Peak **26** was conditionally identified as kaempferol with a deprotonated molecule at 285.0395 with the characteristic fragment ions at *m/z* 257, 213, and 187 [19]. Peak **30** was identified as kaempferol-3-*O*-rhamnoside based on the removal of rhamnosyl moiety. Peak **34** was assigned as rhamnocitrin 3-*O*-sulfate, and fragmentations indicated that the removal of a sulfate group (80 u) yielded a rhamnocitrin aglycone moiety (*m/z* 299), with further loss of a methyl radical (15 u) giving the fragment ion at *m/z* 285.

Apart from quercetin, myricetin, catechin, and kaempferol derivatives, other flavonoid signals could be detected in *A. elliptica* leaf extract. Peak **4** was tentatively identified as 5,7-dimethoxyflavone, with a deprotonated molecule at *m/z* 281.0331 and fragmentation ions at *m/z* 219 and 201, due to the loss of two methoxy groups and one hydroxyl group. Peak **8** showed a deprotonated molecule at *m/z* 549.1448 and fragmentation ion at *m/z* 269, suggesting the presence of a formononetin unit. Peak **10** was tentatively identified as 5,2′,4′,5′-tetrahydroxy-3-(3-hydroxy-3-methylbutyl)-6″,6″-dimethylpyrano[2″,3″:7,8]flavone, commonly named KB-2, with a deprotonated molecule at *m/z* 453.1605 and fragment ion at *m/z* 367 due to the elimination of C_5_H_11_O [23].

Furthermore, theasinensin A (Peak **11**) could be detected with a deprotonated molecule at *m/z* 913.1451 and fragment ions at *m/z* 743 and 575 due to the loss of gallic acid moieties (169 u) [24]. Peaks **12**, **27**, and **32** were respectively assigned as apigenin 7-sulfate, luteolin 7-sulfate, and isorhamnetin 3-sulfate (persicarin), with the elimination of a sulfate moiety (80 u), yielding the apigenin fragment ion at *m/z* 270, luteolin fragment ion at *m/z* 285, and isorhamnetin fragment ion at *m/z* 315 [25,26]. Several significant fragment ions were used to distinguish flavone sulfate (peak **27**) from flavonol sulfate (peak **34**) derivatives at *m/z* 105, 133, and 178 which are characteristic of flavone fragmentation. Furthermore, due to the additional hydroxyl group, fragment ions at *m/z* 110, 151, 162, and 211 are characteristic of flavonol [27]. Peak **29** was conditionally detected as 7,2′-dihydroxyflavone 7-glucoside, with a deprotonated molecule at *m/z* 415.1962 and a fragment ion at *m/z* 252 due to the loss of a glucosyl moiety (162 u). Peak **35** was tentatively assigned as isoscutellarein 4′-methyl ether 8-(2″-sulfatoglucoside) with the elimination of sulfate and glucosyl moieties, which resulted in fragment ions at *m/z* 461 and 299. Thonningianin B (peak **42**) could be identified with a deprotonated molecule at *m/z* 721.3632 and fragment ion at *m/z* 569 due to the elimination of C_7_H_4_O_4_. Moreover, gossypetin 8-glucoside-3-sulfate, 6-chlorocatechin, acerosin, and cycloheterophyllin could also be detected in *A. elliptica* leaf extract, and they were assigned as peaks **45**, **49**, **50**, and **53**, respectively. Interestingly, few metabolites that were reported in genus *Ardisia* could be tentatively identified in the currently studied leaf extract, including flavonoids, triterpenoids, benzoquinones, fatty acid derivatives, phenolic lipids, and phenol ester. Oxycoccicyanin or peonidin-3-glucoside was assigned as peak **9** with the [M − 2H]^−^ ion at *m/z* 461.1610 and a fragment ion at *m/z* 300 due to the elimination of a glucosyl moiety. Reported triterpenoids could be detected in the current leaf extract, including ardisianoside D (peak **40**) with a deprotonated molecule at *m/z* 883.4165. The fragment ion at *m/z* 456 could be observed due to the loss of arabinose, xylose, and glucose moieties [28].

Furthermore, peak **46** was assigned as friedelan-3-one with a deprotonated molecule at *m/z* 425.2304, consistent with reported data at *m/z* 271 and 245 due to the loss of C_11_H_20_ and C_13_H_26_, respectively [29]. Alpha-amyrin (peak **58**) could also be detected based on fragment ions of a dehydrated deprotonated molecule at *m/z* 407, with residues of C_21_H_33_, C_19_H_29_, and C_16_H_24_ at *m/z* 285, 257, and 216 respectively, which corresponded with the reported MS/MS data of alpha-amyrin [30]. Several benzoquinone derivatives were also tentatively identified in *A. elliptica* leaf extract at peaks **41, 44, 47, 54, 57, 59**, and **60**. Ardisianone A (peak **41**) was conditionally identified with a deprotonated molecule at *m/z* 345.1830, as well as fragmentation ions at *m/z* 306 (loss of C_3_H_7_), 292 (loss of C_4_H_9_), and 192 [31]. Peak **44** showed a deprotonated molecule at *m/z* 293.2114 with fragment ions at 275 (loss of H_2_O), as well as 155 and 141 (benzoquinone unit with 2 hydroxyl groups), which are characteristic of embelin [32]. Ardisiaquinones which were previously reported in *Ardisia* genus could be detected in the current leaf extract. Peak **47** was conditionally identified as ardisiaquinone G with a deprotonated molecule at *m/z* 571.2880, as well as characteristic fragment ions at *m/z* 530 and 487 due to the loss of two acetyl units (84 u); the fragment ion at *m/z* 390 implied the two fully substituted benzoquinone rings present in ardisiaquinone G [33]. Ardisiaquinone D was assigned as peak **57** with a deprotonated molecule at *m/z* 541.3524 and product ions at *m/z* 526 and 511 due to the loss of two methyl groups, while *m/z* 359 and 183 showed the presence of a benzoquinone ring. The demethylation of metabolite 57 yielded ardisiaquinone A (peak **59**) with a deprotonated molecule at *m/z* 527.3369, as well as fragment ions at *m/z* 514 and 499, due to the elimination of two methyl groups, whereas *m/z* 191, 165, and 151 implied the presence of benzoquinone rings [34]. Peak **60** was identified as ardisiaquinone J with a deprotonated molecule at *m/z* 529.3524 and fragmentation patterns of *m/z* 514 (loss of methyl group), 499 (loss of methoxy group), and 347 (loss of one benzoquinone ring), which corresponded to the reported MS/MS data of ardisiaquinone J [35].

Peak **54** was assigned as maesaquinone which can be found in the Primulaceae family. Maesaquinone showed a deprotonated molecule at *m/z* 417.2634 with fragment ions at *m/z* 401 (loss of hydroxyl group), 335 (loss of C_6_H_11_), and 193, suggesting the presence of a benzoquinone ring [34]. Ardisiphenol B (peak **38**) was one of the phenol esters with a deprotonated molecule at *m/z* 375.1778 and fragmentation patterns of *m/z* 333 (elimination of acetyl group) and 207 (elimination of acetyl group and C_9_H_17_), which aligned with previously reported MS/MS data of this metabolite [36]. Peak **39** was conditionally identified as ardisinol II with a deprotonated molecule at *m/z* 289.1803, as well as characteristic fragmentation ions at *m/z* 245 (loss of C_3_H_7_), 161, 148, and 123 (loss of C_12_H_23_), which was consistent with reported MS/MS data [37].

Cornudentanone (peak **48**) was conditionally identified with a deprotonated molecule at *m/z* 377.2329 and fragmentation patterns at *m/z* 359, 335 (loss of acetyl group), 316 (loss of ester group, C_2_H_3_O_2_), and 152, suggesting the presence of a benzoquinone ring. Peak **51** showed a deprotonated molecule at *m/z* 385.2741 and fragment ions at *m/z* 268 and 176, suggesting the presence of a hydroxyl benzoyl ion with a C_6_H_12_ aliphatic chain, and *m/z* 153 (presence of trihydroxyl benzoyl ion), which was then tentatively identified as ardisinone E [38]. Alkenylresorcinols such as bilobol (peak **52**) could also be identified with a deprotonated molecule at *m/z* 317.2477, as well as fragment ions at *m/z* 300 (loss of hydroxyl moiety), 231 (loss of C_6_H_13_ aliphatic chain), 192 (loss of C_9_H_17_ aliphatic chain), 178, and 151, which aligned with previously reported MS/MS data [39]. Peak **62** was tentatively assigned as ardisenone with fragmentation patterns at *m/z* 316 (loss of C_10_H_11_O_3_), 278 (loss of C_13_H_16_O_3_), 205 (loss of C_18_H_25_O_3_), 181, and 169 with a deprotonated molecule at *m/z* 495.2625 [40].

Phenolic derivatives such as monogalloylglucose could be observed in *A. elliptica* leaf extract. Monogalloylglucose (peak **2**) could be detected with a deprotonated molecule at *m/z* 331.0662 and fragment ion at *m/z* 169, suggesting the presence of a gallic acid unit due to the loss of a glucosyl moiety [19]. Peak **1** was identified with a deprotonated molecule at *m/z* 191.0184 and fragment ions at *m/z* 173, 129, and 111 (loss of hydroxyl and carbon dioxide moieties), which are characteristic of citric acid [41]. 1,5-Dibutyl methyl hydroxycitrate (peak **61**) could be identified with a deprotonated molecule at *m/z* 333.2426 and fragmentation patterns at *m/z* 279 (loss of C_4_H_9_), 186, and 134 [42]. Peak **55** showed a deprotonated molecule at *m/z* 331.2267 with fragment ions at *m/z* 313 (loss of hydroxyl moiety) and 287 (loss of CHO_2_), which was tentatively identified as Gibberellin A4. Moreover, berberine was conditionally assigned as peak **56** which showed a deprotonated molecule at *m/z* 335.1342 with fragment ions at *m/z* 332, 317 (loss of hydroxyl moiety), and 279, which showed the transition of C_20_H_18_NO_4_ to C_16_H_24_NO_3_ [43]. Fatty acid derivatives could also be detected at peaks **36** and **37** with signature fragmentation patterns at *m/z* 229, 211, and 171 [41]. With the aid of phytochemical characterization, it provides a view of the antioxidant and anti-α-glucosidase potential of 70% ethanolic *A. elliptica* leaf extract. A large number of flavonoids such as quercetin, catechin, and kaempferol derivatives present in the extract were proven to play a role as antioxidant and anti-α-glucosidase agents [44,45]. Other types of compounds such as triterpenoids and benzoquinones might also contribute synergistically to the overall biological activities.

## 3. Materials and Methods

### 3.1. Chemicals and Reagents

Folin–Ciocalteu reagent and absolute ethanol were purchased from Merck (Darmstadt, Germany). Sodium carbonate, DPPH, α-glucosidase enzyme, *p*-nitrophenyl-α-d-glucopyranose (PNPG), glycine, phosphate-buffered saline, sodium nitroprusside (SNP), and other standard compounds used, including gallic acid and quercetin, were purchased from Sigma-Aldrich Co. (St. Louis, MO, USA). LC-MS-grade methanol, purified water, formic acid (FA), and dimethyl sulfoxide (DMSO) were supplied by Fisher Scientific (Geel, Belgium).

### 3.2. Plant Collection and Sample Preparation

Leucaena leucocephala (MFI 0079/19), Muntingia calabura (SK 3345/18), Spondias dulcis (MFI 0065/19), Annona squamosa (SK 2956/16), Ardisia elliptica (MFI 0054/19), Cynometra cauliflora (SK 1757/2011), Ficus auriculata (MFI 0146/19), and Averrhoa bilimbi (MFI 0139/19) were collected from Sri Serdang, Selangor in March 2018. The plants used in this study were identified by Dr. Mohd Firdaus Ismail, an in-house botanist at the Biodiversity Unit, Institute of Bioscience, Universiti Putra Malaysia. The leaves were separated from the stem and cleaned of any impurities with a clean tissue. The leaves were subjected to air drying treatment at room temperature in the shade until a constant weight was achieved [46]. The dried leaves were then ground into fine powder using a laboratory blender (Waring Commercial, Torrington, CT, USA) and stored at 4 °C for further analysis.

### 3.3. Sample Extraction

Sample extraction was done using the method of Mediani et al. [46] with slight modification. Briefly, 10 g of the plant sample was weighed and soaked in 100 mL of 70% ethanol and subjected to sonication at a controlled temperature (26–40 °C) using a Thermo-10D Ultrasonic Cleaner (Fisher Scientific, Waltham, MA, USA) for 1 h. The mixture was then filtered using Whatman filter paper No. 1 (GE Healthcare, Pittsburgh, PA, USA), and the crude extract was vacuum-evaporated using a rotary evaporator. The process was repeated twice using the residue of filtration to achieve maximum yield. The crude extracts were weighed and freeze-dried in a ScanVac CoolSafe Freeze dryer TM (Labogene, Lynge, Denmark). Freeze-dried samples were stored at 4 °C until further analysis.

### 3.4. Total Phenolic Content (TPC) Determination

The TPC was determined using the modified method by Zhang et al. [47]. A volume of 20 μL gallic acid, which was used as the standard, was mixed with 100 μL of Folin-Ciocalteu reagent in a 96-well plate. The mixture was left for 5 min until the addition of 80 μL of 7.5% sodium carbonates to each well. The plate was then incubated in the dark at room temperature for 30 min. The absorbance was measured at 750 nm using a Tecan Infinite F200 micro-plate reader (Tecan Group Ltd., Männedorf, Switzerland) in triplicate measurement. The same procedure was repeated using test samples to replace the standard. The gallic acid standard curve obtained was used to calculate the phenolic content of leaf extracts, which was expressed as mg of gallic acid equivalent per gram of crude extract (mg·GAE/g).

### 3.5. DPPH Free Radical Scavenging Assay

The assay was done using the method of Wan et al. [48] in a 96-well plate using serial dilutions of 50 µL of test sample (330–40 µg/mL). A volume of 100 µL of DPPH was then mixed with the serial diluted test samples. Then, the mixture was incubated for 30 min in the dark at room temperature. The absorbance was measured at 515 nm using a micro-plate reader in triplicate measurement. The scavenging capacity (SC) of the leaf extract was calculated as SC% = [(A_0_ − A_s_)/A_0_] × 100, where A_0_ is the absorbance of reagent blank, whereas A_s_ is the absorbance of test samples. The result was conveyed as IC_50_ value, signifying the concentration of sample required to scavenge 50% of DPPH free radicals. Quercetin (positive control) was used in this assay.

### 3.6. Nitric Oxide (NO) Scavenging Assay

Based on the method used by Tsai et al. [49], the NO scavenging assay was done on a 96-well plate. Then, 60 µL of 10 mM SNP in phosphate-buffered saline was mixed with 60 µL of test samples (330–5.16 µg/mL) in a 96-well plate and incubated for 150 min. Then, 60 µL of freshly prepared Griess reagent was mixed with the test samples before the absorbance was measured at 550 nm using a micro-plate reader. Gallic acid was used as positive control, and the results were reported as IC_50_.

### 3.7. Anti-α-Glucosidase Assay

Anti-α-glucosidase assay was done as previously reported by Lee et al. [19]. The enzyme reaction was achieved using PNPG as the substrate and α-glucosidase enzyme, which were dissolved in 50 mM sodium phosphate buffer. Quercetin was used as the positive control. The test samples were prepared at concentrations of 500, 30, 25, 15, 3, and 1 µg/mL in accordance with the preliminary data obtained through screening analysis. Then, six serial dilutions were done. Thereafter, 10 µL of α-glucosidase enzyme was pipetted into a mixture of 10 µL of test sample and 130 µL of 30 mM phosphate buffer in a 96-well micro-plate. The negative control was prepared by substituting the sample with solvent, whereas blank solvent and blank sample were prepared by 140 µL of 30 mM sodium phosphate buffer with 10 µL of solvent, and 140 µL of 30 mM sodium phosphate buffer with 10 µL of test sample, respectively. Then, the mixture was incubated for 5 min at room temperature. The reaction was started by the adding 50 µL of PNPG substrate into each well of test sample, as well as into the negative and positive controls, while the remaining wells received 50 µL of 30 mM sodium phosphate buffer. The mixture was then incubated for 15 min at room temperature. The reaction was ceased with the addition of 50 µL of 2 M glycine (pH 10). The absorbance was then measured at 405 nm using a micro-plate reader in triplicate measurement. The percentage inhibition of the test sample was calculated as % = [(a_n_ − a_s_)/a_n_] × 100%, where a_n_ is the absorbance difference value between negative control and the blank, and a_s_ is the absorbance difference value between the sample and the blank. The result was expressed as IC_50_ value in µg/mL.

### 3.8. UHPLC–MS/MS Analysis

Based on the method used by Abd Ghafar et al. [50], the UHPLC-MS/MS analysis slight adjustment was done. The UHPLC-MS/MS spectrum of the active extract was acquired using a Thermo Scientific^TM^ Q Exactive^TM^ Hybrid Quadrupole-Orbitrap mass spectrometer equipped with an electrospray ionization (ESI) source coupled with an auto-sampler and surveyor UHPLC binary pump (Thermo Fisher Scientific, Bremen, Germany). Phytochemical separation was done using an Acquity UPLC HSS T3 column (1.8 µm, 2.1 × 150 mm). The mobile phase used in the separation was LC-MS-grade water (solvent A) and acetonitrile (solvent B), each consisting of 0.1% FA. The programmed gradient was initiated with 5% to 100% solvent B from 0.5 to 30 min, and the solvent system was delivered at a flow rate of 0.4 mL/min. The sample was prepared in 10 mg/mL with an injection volume of 2 µL. Negative ion mode was done in full scan mass spectra acquisition from 150–1500 *m/z* with collision-induced dissociation (CID) energy of 30%. The mass spectra were collected and processed using Thermo Xcalibur Qual Browser software 4.0 (Thermo Fisher Scientific Inc., Waltham, MA, USA).

### 3.9. Statistical Analysis

The results of TPC, DPPH and NO scavenging, and anti-α-glucosidase activities were shown as means of three replicates ± standard deviation. One-way ANOVA with Tukey’s post hoc test was done to evaluate the significant effect of the factors at a confidence level of 95%. MS Excel (Microsoft, Redmond, WA, USA) and Minitab 17 (Minitab Inc., State College, PA, USA) software was used in the statistical calculation and Pearson correlation analysis.

## 4. Conclusions

The results of the current study illustrated that *Ardisia elliptica*, *Muntingia calabura*, and *Cynometra cauliflora* exhibited strong antioxidant and anti-α-glucosidase activities. *A. elliptica* showed the most potent activity among all tested plants. A total of 62 metabolites were tentatively characterized in *A. elliptica* 70% ethanolic leaf extract including flavonoids, benzoquinones, and triterpenoids which might contribute to the significant biological activities. To the best of our knowledge, this study provides the first detailed metabolite profile of *A. elliptica* by using UHPLC-ESI-Orbitrap MS. The findings in this work suggest that the leaves of *A. elliptica* could serve as a potential natural source of antioxidant and anti-diabetic agents. However, extensive studies are required to examine the safety and efficacy of their pharmacological properties for the utilization of alternative remedies for disease, particularly diabetes.

## Figures and Tables

**Table 1:** Total phenolic content (TPC), 2,2-diphenyl-1-picrylhydrazyl (DPPH) and nitric oxide (NO) free radical scavenging, and anti-α-glucosidase activities of the extracts. IC_50_—half maximal inhibitory concentration; GAE—gallic acid equivalent.

Leaves Extracts/Standard	Total Phenolic Content (mg GAE/g Sample)	IC_50_ Value (µg/mL)		
		DPPH Free Radical Scavenging Activity	NO Free Radical Scavenging Activity	Anti-α-Glucosidase Activity
*Leucaena leucocephala*	175.75 ± 3.48 ^d^	8.67 ± 0.29 ^d^	65.57 ± 4.57 ^b^	6.62 ± 0.19 ^c^
*Muntingia calabura*	172.32 ± 3.39 ^d^	4.67 ± 0.21 ^c^	59.40 ± 3.39 ^b^	0.51 ± 0.01 ^a^
*Spondias dulcis*	50.90 ± 0.69 ^f^	14.22 ± 0.82 ^e^	301.66 ± 23.06 ^f^	45.52 ± 2.18 ^e^
*Annona squamosa*	199.62 ± 7.40 ^c^	5.00 ± 0.20 ^c^	109.02 ± 3.18 ^c^	3.59 ± 0.18 ^b^
*Ardisia elliptica*	253.10 ± 1.19 ^b^	2.17 ± 0.08 ^a^	49.43 ± 0.18 ^b^	0.29 ± 0.01 ^a^
*Cynometra cauliflora*	344.17 ± 10.80 ^a^	2.88 ± 0.05 ^ab^	118.62 ± 3.44 ^cd^	0.90 ± 0.02 ^a^
*Ficus auriculata*	167.15 ± 2.04 ^d^	5.06 ± 0.35 ^c^	169.65 ± 1.53 ^e^	0.36 ± 0.02 ^a^
*Averrhoa bilimbi*	97.50 ± 3.46 ^e^	16.80 ± 0.04 ^f^	134.33 ± 2.46 ^d^	26.91 ± 0.58 ^d^
Quercetin	-	3.55 ± 0.28 ^b^	15.85 ± 0.58 ^a^	6.62 ± 0.03 ^c^
Gallic acid	-	-	15.41 ± 0.63 ^a^	-

The results are expressed as means ± standard deviation. Means with different superscript letters are significantly different (*p* < 0.05) among leaf extracts. “-” indicates that the particular activities were not measured because of irrelevance to the compound.

**Table 2:** Mass spectral characteristics and tentative identification of compounds present in 70% ethanolic leaves extract of *A. elliptica*.

Peak No.	Retention Time, Min	Exact Mass	Deprotonated Molecule [M − H]^−^ (*m/z*)	Delta	MS/MS Fragment Ions	Tentative Identification	Molecular Formula
1	1.00	192.0197	191.0184	0.0013	173.0080, 129.0180, 111.0074, 87.0073	Citric acid	C_6_H_8_O_7_
2	1.19	332.0671	331.0662	0.0009	312.1069, 271.0454, 241.0342, 211.0238, 169.0130	Monogalloylglucose	C_13_H_16_O_10_
3	3.14	452.4087	451.3394	0.0693	302.5710, 289.0708, 210.2854, 151.2638	(+)-Catechin 6-C-glucoside	C_21_H_24_O_11_
4	3.31	282.0817	281.0331	0.0486	239.1548, 219.1387, 207.1384, 201.1277, 165.0904	5,7-Dimethoxyflavone	C_17_H_14_O_4_
5	3.40	282.0817	281.0329	0.0488	239.1548, 219.1387, 207.1384, 201.1277, 165.0904	5,7-Dimethoxyflavone isomer	C_17_H_14_O_4_
6	3.45	282.0817	281.0328	0.0489	239.1548, 219.1387, 207.1384, 201.1277, 165.0904	5,7-Dimethoxyflavone isomer	C_17_H_14_O_4_
7	3.70	762.1571	761.1343	0.0228	610.1257, 301.0352, 169.0131	Quercetin 3-*O*-(2″-*O*-galloyl)-rutinoside	C_34_H_34_O_20_
8	3.80	550.1402	549.1448	0.0046	429.1028, 369.0819, 339.0715, 309.0611, 269.0662	Formononetin 7-*O*-(2″-*p*-hydroxybenzoylglucoside)	C_29_H_26_O_11_
9	4.14	463.1168	461.1610 *	0.0442	314.0427, 300.1083, 287.0562, 255.0286	Oxycoccicyanin/Peonidin-3-glucoside	C_22_H_23_O_11_
10	4.34	454.1555	453.1605	0.0050	386.9793, 367.0700, 301.0338, 284.0323, 176.0435	KB-2/5,2″,4′,5′-Tetrahydroxy-3-(3-hydroxy-3-methylbutyl)-6″,6″-dimethylpyrano[2″,3″:7,8]flavone	C_25_H_26_O_8_
11	4.57	914.1469	913.1451	0.0018	761.1329, 743.1249, 609.1331, 591.1135, 575.0852, 453.0853	Theasinensin A	C_44_H_34_O_22_
12	4.79	350.0024	349.0591	0.0567	269.6351, 241.0011, 227.0375, 152.0433	Apigenin 7-sulfate	C_15_H_10_O_8_S
13	4.95	290.0790	289.1802	0.1012	245.0812, 203.0703, 179.0335, 137.0230, 123.0437	Catechin	C_15_H_14_O_6_
14	5.02	458.0776	457.0766	0.0010	331.0454, 305.0666, 287.0562, 269.0456, 193.0132, 169.0131	(−)-epigallocatechin-3-gallate	C_22_H_18_O_11_
15	5.61	756.2040	755.2025	0.0015	489.1044, 301.0315, 300.0270, 271.0243, 255.0294, 178.9978	Quercetin 3-*O*-(2,6-di-*O*-rhamnosylglucoside)	C_33_H_40_O_20_
16	5.71	480.0831	479.0816	0.0015	316.0219, 287.0189, 271.0241, 178.9979, 151.0025	Myricetin-3-*O*-glucoside	C_21_H_20_O_13_
17	5.76	626.1410	625.1396	0.0014	478.0751, 317.0288, 316.0212, 271.0243, 178.9976	Myricetin-3-*O*-rutinoside	C_27_H_30_O_17_
18	5.93	596.1305	595.1293	0.0012	463.0802, 301.0349, 300.0271, 283.0230, 271.0244, 178.9975	Quercetin 3-lathyroside	C_26_H_28_O_16_
19	6.11	450.0726	449.0713	0.0013	316.0220, 287.0198, 271.0241, 178.9975	Myricetin-3-*O*-arabinoside	C_20_H_18_O_12_
20	6.32	610.1461	609.1451	0.0010	463.0797, 447.0925, 301.0349, 300.0247	Quercetin 3-*O*-α-l-rhamnoside-7-*O*-β-d-glucoside	C_27_H_30_O_16_
21	6.38	610.1461	609.1450	0.0011	301.0339, 300.0269, 271.0244, 178.9973, 151.0026	Quercetin-3-*O*-rutinoside (Rutin)	C_27_H_30_O_16_
22	6.45	464.0882	463.0869	0.0013	316.0218, 287.0193, 178.9977, 151.0026	Myricetin-3-*O*-rhamnoside	C_21_H_20_O_12_
23	6.47	381.9922	380.9911	0.0011	301.0349, 300.0247, 283.0245, 271.0238, 257.0445, 229.0496, 193.0133	Quercetin 3-*O*-sulfate	C_15_H_10_O_10_S
24	6.66	464.0882	463.0875	0.0007	301.0339, 300.0270, 271.0244, 255.0293, 178.9976, 151.0024	Quercetin-3-*O*-glucoside	C_21_H_20_O_12_
25	6.95	434.0776	433.0768	0.0008	301.0346, 300.0271, 271.0243, 255.0291, 178.9982, 151.0023	Quercetin-3-*O*-arabinoside	C_20_H_18_O_11_
26	7.18	286.0405	285.0395	0.0012	257.0450, 213.0545, 187.0391, 163.0021	Kaempferol	C_15_H_10_O_6_
27	7.19	365.9973	364.9961	0.0010	285.0400, 267.0294, 255.0291, 241.0501, 229.0500, 213.0548, 178.4121, 133.0279, 105.6355	Luteolin 7-sulfate	C_15_H_10_O_9_S
28	7.44	448.0933	447.0917	0.0016	301.0339, 300.0271, 271.0243, 255.0291, 178.9975, 151.0024	Quercetin-3-*O*-rhamnoside	C_21_H_20_O_11_
29	7.64	416.1035	415.1962	0.0927	252.1096, 238.9105, 177.2131, 123.0804	7,2′-Dihydroxyflavone 7-glucoside	C_21_H_20_O_9_
30	8.31	432.0984	431.0970	0.0014	285.0393, 284.0321, 255.0293, 227.0341	Kaempferol-3-*O*-rhamnoside	C_21_H_20_O_10_
31	8.66	458.0776	457.0766	0.0010	331.0454, 305.0666, 287.0562, 269.0456, 193.0132, 169.0131	Epigallocatechin-3-gallate isomer	C_22_H_18_O_11_
32	9.15	396.0079	395.0064	0.0015	315.0607, 272.0317, 259.0608, 151.0027	Persicarin/Isorhamnetin 3-sulfate	C_16_H_12_O_10_S
33	9.57	302.0354	301.0348	0.0006	273.0403, 178.9974, 151.0024, 121.0281	Quercetin	C_15_H_10_O_7_
34	10.00	380.0129	379.0117	0.0012	299.0555, 284.0321, 257.0403, 243.0656, 228.0399, 211.0385, 162.5436, 151.0027, 110.0001	Rhamnocitrin 3-*O*-sulfate	C_16_H_12_O_9_S
35	10.37	542.0658	541.0644	0.0014	461.1082, 314.0426, 299.0188, 271.0243, 256.0363, 158.7938	Isoscutellarein 4′-methyl ether 8-(2′-sulfatoglucoside)	C_22_H_22_O_14_S
36	11.20	328.2177	327.2171	0.0006	229.1440, 211.1331, 171.1015	Trihydroxy octadecadienoic acid	C_18_H_32_O_5_
37	11.98	328.2177	327.2171	0.0006	229.1440, 211.1331, 171.1015	Trihydroxy octadecadienoic acid isomer	C_18_H_32_O_5_
38	13.78	376.2541	375.1778	0.0763	333.6364, 330.1770, 329.1730, 307.1919, 235.1334, 207.0993	Ardisiphenol B	C_23_H_36_O_4_
39	14.49	290.2173	289.1803	0.0370	245.1902, 161.9148, 148.7701, 123.0794	Ardisinol II	C_19_H_30_O_2_
40	16.00	884.5061	883.4165	0.0896	837.4141, 559.1864, 456.2514, 397.1332, 277.2172	Ardisianoside D	C_46_H_76_O_16_
41	16.37	346.2497	345.1830	0.0667	306.9802, 292.2949, 192.5377	Ardisianone A	C_22_H_34_O_3_
42	16.54	722.1410	721.3632	0.2222	675.3585, 569.1615, 415.1444, 400.9850, 305.0875, 277.2165	Thonningianin B	C_35_H_30_O_17_
43	16.96	722.1410	721.3631	0.2221	675.3580, 569.1614, 415.1447, 400.9853, 305.0875, 277.2166	Thonningianin B isomer	C_35_H_30_O_17_
44	18.14	294.1758	293.2114	0.0356	275.2011, 155.1072, 141.1270, 127.1115, 121. 1009	Embelin	C_17_H_26_O_4_
45	18.67	560.0399	559.1269	0.0870	354.6981, 286.8783, 228.8837, 121.6282	Gossypetin 8-glucoside-3-sulfate	C_21_H_20_O_16_S
46	19.33	426.3789	425.2304	0.1485	271.0612, 245.0811, 203.0705, 177.0179, 151.0386	Friedelan-3-one	C_30_H_50_O
47	20.00	572.2621	571.2880	0.0259	530.8586, 487.1684, 391.2236, 255.2324, 241.0111, 223.0012	Ardisiaquinone G	C_31_H_40_O_10_
48	22.76	378.2334	377.2329	0.0005	359.2229, 335.2515, 316.2326, 152.0106	Cornudentanone	C_22_H_34_O_5_
49	23.12	324.0328	323.1162	0.0834	279.2323, 265.8335, 216.0093, 184.0194	6-Chlorocatechin	C_15_H_13_ClO_6_
50	23.80	360.0772	359.0612	0.0160	317.2494, 315.2661, 245.0694, 211.2592	Acerosin	C_18_H_16_O_8_
51	24.48	386.2093	385.2741	0.0648	268.6892, 176.3903, 153.4738	Ardisinone E	C_23_H_30_O_5_
52	25.02	318.2558	317.2477	0.0081	300.0236, 231.3264, 192.0054, 178.9974, 151.0025	Bilobol	C_21_H_34_O_2_
53	25.24	502.1919	501.3211	0.1292	486.2979, 473.2810, 456.2825, 443.2783, 435.2527	Cycloheterophyllin	C_30_H_30_O_7_
54	26.18	418.3083	417.2634	0.0449	401.6784, 375.2524, 335.2505, 308.5951, 193.0859	Maesaquinone	C_26_H_42_O_4_
55	26.33	332.1551	331.2267	0.0716	313.2373, 287.2391, 254.8929, 225.6182, 213.1681	Gibberellin A4	C_19_H_24_O_5_
56	26.54	336.1163	335.1342	0.0179	332.2440, 317.2474, 305.2462, 279.2698, 230.0207	Berberine	C_20_H_18_NO_4_
57	26.80	542.2879	541.3524	0.0645	526.3293, 511.3054, 493.2956, 359.2590, 183.6019	Ardisiaquinone D	C_31_H_42_O_8_
58	26.93	426.3861	425.2300	0.1489	407.0769, 381.0991, 339.0862, 257.0452, 216.0410, 167.2586	Alpha-amyrin	C_30_H_50_O
59	27.05	528.2723	527.3369	0.0646	514.3290, 499.3061, 191.0708, 165.0543, 151.0386	Ardisiaquinone A	C_30_H_40_O_8_
60	27.08	530.2952	529.3524	0.0572	514.3292, 499.3058, 481.2948, 453.2976, 347.2579, 225.7453	Ardisiaquinone J	C_30_H_42_O_8_
61	28.09	334.1555	333.2426	0.0871	279.2678, 186.7317, 134.0364	1,5-Dibutyl methyl hydroxycitrate	C_15_H_26_O_8_
62	28.37	496.2824	495.2625	0.0199	316.1812, 278.8960, 205.1232, 181.0499, 169.0134	Ardisenone	C_30_H_40_O_6_

* Represented [M − 2H]^−^ ion was observed.
